# The COVID-19 pandemic, proper science and everything else

**DOI:** 10.7189/jogh.11.01002

**Published:** 2021-04-03

**Authors:** Igor Rudan

**Affiliations:** Centre for Global Health, the Usher Institute, The University of Edinburgh, Scotland, UK

The information, the science, the media and the general public didn’t show much compatibility during the COVID-19 pandemic. The international scientific community, generally speaking, has done very well in the fight against the COVID-19 pandemic during 2020. However, from the perspective of the media and the general public, this could not always be seen clearly enough or appreciated sufficiently.

Using the latest technologies, proper science has been responding quite well from month to month to all the challenges of this pandemic. It has been fulfilling its tasks, slowly offering one answer after another to very many unknowns and open questions, and patiently reaching its goals. Therefore, scientists are now the only tiny fraction of people with a chance to get us all out of this difficult crisis through implementation of vaccines and new drugs. The actual contribution of science will be understood much better in the books that will be published in the coming years about the COVID-19 pandemic.

Going back to the very beginning of the pandemic, four important factors needed to be distinguished in all events:

1. the availability of credible scientific data and information on the pandemic at all times, which was constantly changing in the early stages [1,2];
2. the scientific community, which was both the primary source and primary user of that information;
3. all of the media and social networks;
4. the general public, which has shown a chronic hunger for information on the pandemic, which was hardly comparable to any event in the history of global media. Temporarily, such interest can occasionally be seen during the Olympics, World Cups and wars, but this pandemic has become a permanent condition.

It should first be understood that these four factors were not homogeneous at any point. First, scientific information was initially very scarce and not always reliable. However, more than 100 000 scientific papers have been peer-reviewed and published to date [3]. As a result, the information we have today is much more reliable. It can no longer be said that we are still unsure of many things. On most issues, the scientific community is in complete agreement.

However, the scientific community is not homogeneous in itself, either. Among the scientists, there are those for whom this field is their speciality and who could hardly make a mistake in interpreting the data that was ever presented to them. However, there were also those for whom epidemics of infectious diseases was not a speciality, but they still engaged in public commentary. They have, unfortunately, too often been sources of confusion and misunderstanding.

To sufficiently understand the COVID-19 crisis, it would take years to study and practice medicine, virology, public health, epidemiology, immunology, infectious diseases, possess enough knowledge on the pharmaceutical industry and health systems, and have some understanding of many other areas of knowledge. Many of those who commented on the pandemic simply did not have all this knowledge. However, part of the population trusted them because they themselves knew even less. They especially believed the scientists, irrespective of their backgrounds, when they heard from them what they actually wanted to hear.

Then, the world of global and national media is not homogeneous, either. There are responsible media outlets, the likes of the New York Times or The Atlantic, or the BBC and the Guardian, where it is hardly possible to read anything that was not well checked and put into context in the correct way using several additional statements from renowned and independent experts [4]. But there were also sources who embraced sensationalism. Those would uncritically publish and disseminate without proper verification just about every new information that the algorithms detected to be spreading the fastest on social media, regardless of its reliability.

And finally, the general public is far from homogeneous. A small part of it is highly educated and critical enough to be able to distinguish the essential differences between the news that were spread, the people who communicated them, and the media and the sources that transmitted them. However, we have also had the opportunity to notice that a significant portion of the international public has not developed this critical ability. Many people on all continents have succumbed to misinformation.

If I have personally learned anything from this pandemic, it is how inhomogeneous the public is everywhere in the world. Whoever enters the public scene and starts talking about anything often enough, loudly enough, and confidently enough, they will gather a part of the public around themselves, no matter what they talked about and what their credentials were. They will be particularly successful if they manage to choose topics that would gather people who are in fear, who don’t have a lot of personal knowledge, who have insufficient resources, and who are being told what they want to hear. This has been seen many times throughout history in times of crisis [5].

In most countries there came a point in this pandemic when it became pointless to try to convince people of anything. It is really difficult to assess what the public in any country in the world really believes in today about COVID-19. The only effective remedy for this chaotic situation was reality itself, which changed on a daily basis. It was the reality that denied rumours and misinformation, one at the time. Scientists and other reasonable people were unable to do this themselves because this was an extraordinary situation.

In extraordinary situations, the voice of reasonable people is usually least heard. It is quite likely that, at the beginning of any deadly war, there were quite a few people who called to reason, but no one remembers them. That is why reality remained a key ally of common sense. It persistently played the most important role in denying wrong hypotheses and theories. Still, even when it did so very clearly, various groups would still find a way to twist it and recycle it in some way that would still seemingly fit their misconceptions yet again.

In such a dynamic and inhomogeneous environment, where people lived for months in an atmosphere of fear and even panic, it is understood that there was a rarely seen noise in the media and social networks in which nothing seemed certain. It seemed possible that literally any acquired understanding or knowledge could change from one day to another. Occasionally, it seemed to the general public that even those who were trained to deal with pandemics were lost. In some phases of the pandemic of COVID-19, they seemed to have communicated one thing and in others something entirely different. Such occasions have certainly damaged the reputation of many scientists and public confidence in science. The public could not have known that pandemics are extremely dynamic events, such as unpredictable wars or tense football matches, in which outcomes are constantly changing and readjustments are often required to reduce losses and increase the chances of a successful ending.

However, as the impression of the noise, chaos and uncertainty grew in the public and the media, a consensus on most issues was growing in the scientific community with each new published scientific paper. This pandemic has shown that the scientific method and the process of acquiring scientific knowledge, whose main features include patience, meticulousness, certainty in the result and caution in assessments, is simply incompatible with the public interest in media coverage of the pandemic; there, it was the speed and attractiveness of the information that ruled, and the media could not always wait for the sufficient level of certainty over each piece of information.

There was an additional problem, which made science and scientists look rather bad from time to time: there were several real, dramatic twists and turns, brought about by unforeseen events or discoveries. It was not possible to present all the necessary details on a topic in real-time. Some events simply proved too complicated to be clarified to the full in the media space, and the general public never properly understood them.

People often complained that some of the best long reads on this pandemic in international media had become too long and detailed for them to read, even though they were always an extremely simplistic representation of a small part of the knowledge that was really required at that point in time to properly understand the complexities of the pandemic.

Fortunately, we have entered a phase of the pandemic of COVID-19 in which science is already clear enough about its truths. The scientific community is united around the vast majority of important issues. From this point, members of the public can choose to either trust proper science and scientists, who will slowly pull us all out of this difficult crisis, or anything else.
